# Cavernous hemangioma of the testis mimicking a testicular teratoma

**DOI:** 10.3892/etm.2013.1086

**Published:** 2013-04-29

**Authors:** BEN LIU, JUN CHEN, JINDAN LUO, FENG ZHOU, CHAOJUN WANG, LIPING XIE

**Affiliations:** Department of Urology, First Affiliated Hospital, College of Medicine, Zhejiang University, Hangzhou, Zhejiang 310003, P.R. China

**Keywords:** testis, hemangioma

## Abstract

In this study we report a case of cavernous hemangioma of the testis, which mimicked a testicular teratoma. A 42-year-old male presented with a left testicular swelling that had arisen suddenly and been present for three months. Scrotal ultrasound revealed a hypoechoic mass with several calcifications in the left testicle. The mass demonstrated blood flow in the color Doppler mode. A radical orchiectomy was performed. Pathological evaluation revealed a testicular cavernous hemangioma with thrombus organization and calcification.

## Introduction

Cavernous hemangiomas are benign vascular tumors that may develop in any part of the body. They are composed of large vessels with dilated lumina and thin walls. The vessels may have abnormal walls and cannot be identified as arterial or venous. Thrombosis and calcification are commonly observed in cavernous hemangiomas.

Testicular cavernous hemangiomas are a rare type of benign testicular tumor and distinguishing them from other common testicular tumors prior to surgery is challenging. The chief presenting symptom of testicular cavernous hemangioma is testicular enlargement. In the current study, we report a case of cavernous hemangioma of the left testis, which mimicked a testicular teratoma and was treated with radical orchiectomy, and provide a review of the literature.

## Case report

A 42-year-old male presented with a sudden episode of left testicular fullness for three months. The patient denied any history of hematuria, fever, scrotal trauma or urinary tract infection. The patient’s past medical history and family history were non-contributory.

**Figure 1.**
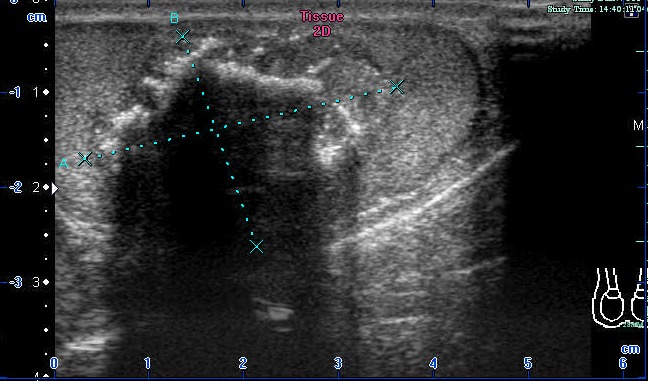
Scrotal ultrasound revealed a roundish, well-demarcated, hypoechoic mass with calcifications in the left testicle.

**Figure 2.**
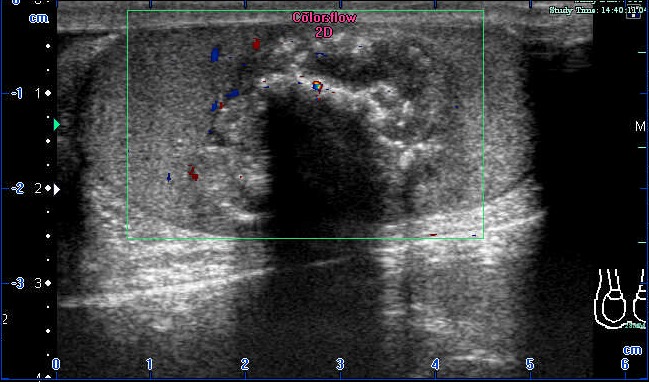
Scrotal ultrasound. The left testicular mass demonstrated blood flow in color Doppler sonography.

**Figure 3.**
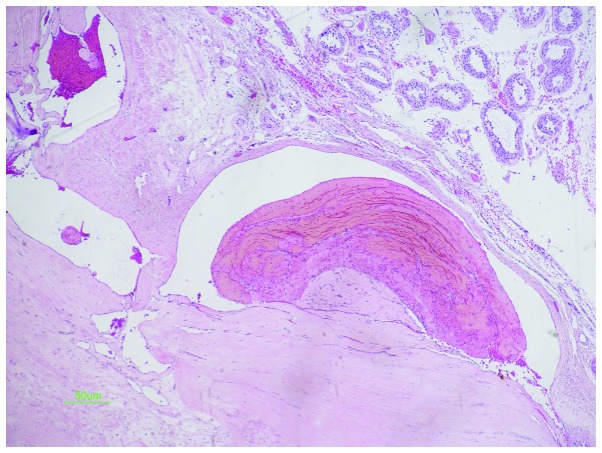
Photomicrograph of a section of the testis reveals a cavernous hemangioma. Original magnification, ×50.

Physical examination revealed a palpable, non-tender, left testicular mass ∼3×2.5 cm in size. The left testis was swollen and stiff. The epididymis and spermatic cord were normal. The patient had a normal blood cell count and urinalysis. Laboratory examinations, including relevant tumor markers, particularly α-fetoprotein and β-human chorionic gonadotrophin, were normal. Scrotal ultrasound revealed a roundish, well-demarcated, hypoechoic mass in the left testicle (Fig. 1) and several calcifications were visible within the mass. The mass demonstrated blood flow in color Doppler sonography (Fig. 2). The patient was diagnosed with a testicular teratoma and a left radical orchidectomy, using an inguinal approach, was performed. However, pathological evaluation of the mass revealed that is was a testicular cavernous hemangioma with thrombus organization and calcifications (Fig. 3). The study was approved by the ethics committee of the First Affiliated Hospital, College of Medicine, Zhejiang University, Zhejiang, China. Written informed patient consent was obtained from the patient.

## Discussion

Cavernous hemangiomas are benign vascular tumors, which may develop in any part of the body. The occurrence of cavernous hemangioma in the testis is rare. The first case of testicular cavernous hemangioma was reported in 1944 and, to date, 23 cases have been reported (1–13). The majority of subtypes of vascular tumors of the testis have been described as cavernous, capillary, histiocytoid and juvenile, with the most common being cavernous hemangioma (2). Hemangiomas most likely arise from the inner layer of the tunica albuginea, which contains blood vessels and lymphatics and sends septa into the testicular parenchyma (9). A hemangioma may extend into the testicular parenchyma by way of these septa. Cavernous hemangiomas are composed of large vessels with dilated lumina and thin walls. They may be composed of vessels whose walls are abnormal and cannot be identified as arterial or venous. Thrombosis and calcification are common in cavernous hemangiomas (9).

The age of onset for testicular cavernous hemangiomas reported in the literature varies from 17 weeks to 77 years (1–13). Testicular enlargement, with or without tenderness, is the chief presenting symptom, which is similar to that of malignant testicular tumors on clinical presentation. However, there are reports of testicular hemangiomas presenting as testicular torsion or associated with testicular infarction (7,11). Distinguishing cavernous hemangioma of the testis from other common testicular tumors prior to surgery is not feasible. Doppler ultrasonography is useful for diagnosing testicular hemangiomas. It demonstrates the nature of the mass and differentiates it from other testicular neoplasms (14). Hemangiomas in sonographs vary from hypoechoic to hyperechoic, or they may be heterogeneous (15). Various sizes of calcification are common, and in this case report we misdiagnosed cavernous hemangioma of the testis as a testicular teratoma. To date, all reported vascular testicular tumors have demonstrated benign behavior, without local recurrence or metastasis (16). Testis-sparing surgery may be performed if intraoperative examination of frozen sections of representative tissue is possible (16).

In conclusion, testicular cavernous hemangioma is rare. When a patient presents with a testicular mass where the ultrasound reveals a mass with calcifications of various sizes and negative tumor marker findings, a diagnosis of testicular cavernous hemangioma should be considered.
